# The Effects of Different Degrees of Leg Length Discrepancy on Vertical Ground Reaction Force in Children and Adults: Treatment Implications

**DOI:** 10.5704/MOJ.2311.010

**Published:** 2023-11

**Authors:** F Mohamed-Saaid, AR Sulaiman, I Munajat, EF Mohd, WN Arifin, R Ghafar

**Affiliations:** 1 Department of Orthopaedics, Universiti Putra Malaysia, Serdang, Malaysia; 2 Department of Orthopaedics, Universiti Sains Malaysia, Kubang Kerian, Malaysia; 3 Unit of Biostatistics and Research Methodology, Universiti Sains Malaysia, Kubang Kerian, Malaysia; 4 Exercise and Sports Science Program, Universiti Sains Malaysia, Kubang Kerian, Malaysia

**Keywords:** length discrepancy, force, height

## Abstract

**Introduction:**

Previous studies on the degree of leg length discrepancy that causes limb biomechanical problems did not differentiate between adults and children. We conducted this study to determine the effects of simulated leg length discrepancy on vertical ground reaction force in children and adults to enable decision-making for intervention in patients with leg length discrepancy for different age groups or heights.

**Materials and methods:**

This cross-sectional study involved male volunteers of children 150cm and adults with 170cm in height. Vertical ground reaction force was measured using a gait analysis study. The first measurement was taken without any leg length discrepancy as a baseline. Subsequently, different amounts of leg length discrepancy were simulated on the left leg with shoe lifts of 2, 3, and 4cm. The measurements were repeated on each volunteer with similar shoe lifts on the right leg. Therefore, 14 volunteers provided simulations of 28 leg length discrepancies for each group. The first and second peaks of vertical ground reaction force were separately analysed. The vertical GRF of a simulated leg length discrepancy was compared with the baseline. Repeated measurement of analysis of variance (ANOVA) within each group was done.

**Results:**

In both groups, the second peak of vertical ground reaction force in the longer leg reduced gradually as the shoe lift increased sequentially from 2 to 3cm and then to 4cm. A discrepancy of 3cm and above was statistically significant to cause a reduction in the vertical GRF on the longer limb in both height groups.

**Conclusion:**

The degree of leg length discrepancy that caused significant changes in second peak ground reaction force in children with 150 and adults with 170cm height population was similar at 3cm. Therefore, the cut-off point for intervention for both groups are similar with additional consideration of future growth in children.

## Introduction

Leg length discrepancy (LLD) is a known cause of multiple orthopaedic problems, including low back pain, osteoarthritis, and stress fracture, secondary to mechanical and functional gait changes1,2. Available treatment options include non-invasive shoe lifts and surgeries such as epiphysiodesis, limb shortening, or lengthening, which are not without complications3. For example, the Ilizarov bone lengthening procedure can cause iatrogenic fractures, pin tract infections, and joint stiffness; the most catastrophic complication can be an injury to neurovascular bundles^3^.

The clinical significance of LLD depends on the amount of discrepancy and the ability of the pelvis and spine to compensate for the inequality^4^. Many studies attempting to quantify significant LLD have been conducted and accept as much as 2 to 3cm discrepancy as compensable by the body^5-8^.

Although many studies have concluded that 3cm is the cut-off amount of LLD that require intervention, none have specified whether same amount of discrepancy should be implied to everyone regardless of the height and age. This is more relevant due to difference adaptation capability between child and adult. A relevant question is whether the percentage of LLD from the patients’ height can influence the effect of LLD on our bodies. For example, a 2cm LLD (1.2%) of 170cm height in adults was not causing significant changes in limb biomechanics, but a 2cm LLD (1.3%) of 150cm height in children might be substantial. This is essential information for the clinician dealing with short-stature patients or children.

LLD is known to be one of the causes of gait asymmetry, the differences in the behaviour of bilateral legs^9^. Gait asymmetry can result in improper weight distribution between limbs^10^ and increased energy expenditure. One way to objectively measure gait asymmetry is by measuring vertical ground reaction force (GRF), the force exerted by the ground on a body in contact^11^. For example, a person standing motionless on the ground exerts a contact force on it (equal to the person’s weight), and at the same time, an equal and opposite GRF is exerted by the ground on the person. Limb loading influences the occurrence of osteoarthritis of the joint^12^. In normal gait, the GRF for both lower limbs should be equal. We selected 170cm height to represent the average adult height and 150cm height to represent children or short-stature people^13^. A difference of 20cm was chosen as it was a visible difference in height. We conducted this study to investigate whether the amount of LLD causing significant vertical GRF changes in children 150cm and adults 170cm in height are different to enable decision-making for intervention in patients with leg length discrepancy from different age groups or heights.

## Materials and Methods

This cross-sectional study was performed on male volunteers with either children with a height (range) of 150 (148–152) cm or adult with 170 (168–172cm) cm, with a normal body mass index (18.5–24.9 kg/m^2^). Volunteers were selected among undergraduate students from Universiti Sains Malaysia and primary school students in Kota Bharu using a simple random sampling method. All volunteers had no complaints regarding gait, could squat fully and had a difference in length of the lower limbs within 0.5cm. Volunteers with neuromuscular diseases such as cerebral palsy, scoliosis, or abnormal joints were excluded. This study was approved by the Human Ethical Committee of the School of Medical Sciences, Universiti Sains Malaysia (USM/)JEPEM/15100350.

The sample size was calculated using G*Power version 3.9.1.2 for the planned repeated measurement of analysis of variance (ANOVA) (within-between interaction)^14^. The effect size was set at 0.7 (Cohen’s) with alpha = 0.05, power = 80%, number of groups = 2, and number of measurements = 4. This resulted in a minimum sample size of 24 subjects per group.

This study was conducted at the Sports Science Laboratory, Universiti Sains Malaysia, for one day to perform the gait analysis study using the 3D Qualisys® motion camera and Qualisys® software. Informed consent was obtained from all volunteers. All volunteers wore tight non-reflective black pants with canvas shoes provided in the laboratory. The true length of both lower limbs was measured using the tape measure method from the anterior superior iliac spine to the tip of the medial malleolus, with the subject in the supine position. Volunteers with no LLD or LLD of less than 0.5cm were chosen to continue the study.

Fifteen reflective markers were applied, one each at the fifth metatarsal head, the heel, the most prominent part of the lateral malleolus, the lateral aspect of the mid-tibial shaft, the lateral femoral epicondyle, the lateral aspect of the mid-femoral shaft, the anterior superior iliac spine and the sacrum, to be captured by a camera. All markers were applied bilaterally except for the sacrum.

Before taking the measurements, all volunteers performed walking trials for 15m on a straight walkway to familiarise themselves with each shoe lift for at least one minute to establish their natural gait. The 400 × 600mm force plate was calibrated before measurements were taken. Immediately after familiarisation, volunteers walked along the straight 6-metres walkway with a single force plate at their self-selected walking speed. The walkway was located approximately equidistant from six cameras placed around the laboratory. The cameras captured the reflective markers when the longer leg stepped on the force plate. Data were gathered from the force plate and cameras at a frequency of 200 hertz. A charge amplifier connected the force plate to a computer for data collection.

The first measurement was taken when volunteers walked on the force plate without any shoe lift; this was repeated three times. A similar procedure was repeated with a shoe lift on one side of the lower limb with sequentially 2, 3, and 4cm lifts. The volunteers were away from the computer screen and could not see the value of the vertical GRF recorded. The shoe raise was stopped at 4cm because a shoe lift of more than that amount was known to cause foot pain^15^. For each level of shoe lift, three walking cycles were recorded within 40 seconds. Each volunteer repeated the procedure with a shoe lift on the other leg. Therefore, 14 volunteers provided a simulation of 28 LLDs. The shoe lifts were made from crepe foam and strapped to the sole of regular canvas shoes with Velcro. We studied 28 LLDs for the 150cm height volunteers and 28 LLDs for 170cm height volunteers.

Using the Qualisys Software, only the first and second peak force vertical GRFs were analysed. Vertical GRFs of the limbs with simulated LLD were compared with the control. Data were entered into SPSS® version 20.0 for analysis. All data were checked and cleaned. Repeated measures ANOVA was used to compare the means between four levels of shoe lift (0, 2, 3, and 4cm) for each of the 150 and 170cm height groups. Whenever the analysis was significant, pairwise comparisons with Bonferroni correction were performed for detailed analysis. The confidence interval (CI) was set at 95%. A P-value of less than 0.05 was considered significant.

## Results

**Fig. 1:**
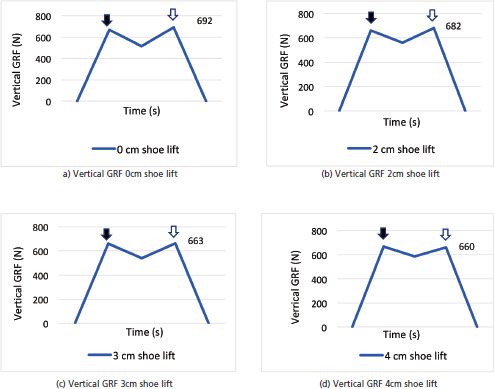
(a) Plot of vertical GRF of longer leg in a participant with 170cm height group at stance phase against time for no lift, (b) 2cm lift, (c) 3cm lift, and (d) 4cm lift.

Notes: The shaded arrow points to the first peak of vertical GRF, and the unshaded arrow points to the second peak of vertical GRF. The plot shows a constant (a) decrease of second peak GRF as the shoe lift (b) increase.

Fourteen volunteers, ranging from 9 to 11 years old, participated in the 150cm height group, and 14 volunteers, ranging from 20 to 27 years old, participated in the 170cm height group. A vertical GRF graph for the longer leg was plotted based on 28 data sets of LLD for each height group, as the simulation of each side was considered independent due to limb dominancy. The graph indicated an ‘M’-shaped curve with two peaks (Fig. 1). During the initial part of the stance phase, the force rapidly increased from zero to form the first peak of the vertical GRF. As the gait cycle progressed, at the mid-stance phase, the force reduced slightly. At the terminal stance phase, the force rapidly increased again, forming the second peak of the vertical GRF (Fig. 1). Finally, the force dropped to zero as the opposite limb took up the body weight. This study analysed the long leg's first and second peak force from the vertical GRF graph.

The mean first peak force for children with 150cm height volunteers was 426.69 N during 0cm shoe lift, 433.27 N during 2cm shoe lift, 425.99 N during 3cm shoe lift, and 430.22 N for 4cm shoe lift on the longer leg. The force differences were not statistically significant when the shoe lift increased gradually from 2 to 4cm (Table I). The mean first peak force for adults with 170cm height volunteers was highest during 0cm of shoe lift (670.62 N) and declined progressively during 2cm (664.58 N) followed by 3cm (657.84 N) of shoe lift. However, it showed a slight increment in 4cm (661.57 N) shoe lift compared to 3cm.

**Table I:** First peak force of vertical ground reaction force on the longer leg for 0, 2, 3 and 4cm shoe lift for children with 150 and adult with 170cm height volunteers.

Height	Shoe lift	First peak force (N) Mean (SD)	F (df1, df2)*	P-value	Partial ŋ^2^
150cm	0cm	426.7 (65.6)	0.567 (3, 81)	0.639	0.021
	2cm	433.3 (65.0)			
	3cm	426.0 (61.0)			
	4cm	430.2 (61.3)			
170cm	0cm	670.6 (89.3)	2.460 (2.141, 57.800)*	0.091	0.084
	2cm	664.6 (91.8)			
	3cm	657.8 (89.9)			
	4cm	661.6 (89.1)			

Note. SD = Standard deviation; F = F statistics; df = degrees of freedom. *Greenhouse-Geisser correction to the degrees of freedom due to violation of the sphericity assumption.

The highest mean second peak force in children with a 150 cm height group occurred during 0cm shoe lift (411.6 N). The force on the longer leg then reduced progressively when the shoe lift was 2cm (398.6 N), followed by 3cm (390.0 N) and further down with the 4cm shoe lift (381.0 N). The analysis showed a significant difference in the mean second peak force of vertical GRF between the four levels of shoe lifts (Table II). Pairwise comparisons showed significant reductions in the force on the longer leg between 0 and 3cm shoe lift and between 0 and 4cm shoe lift, but the difference was not significant between 0 and 2cm shoe lift (Table III).

**Table II:** Second peak force of vertical ground reaction force on the longer leg for 0, 2, 3 and 4cm discrepancy for children with 150 and adult with 170cm height volunteers (n = 28 each).

Height	Shoe lift	Second peak force (N) Mean (SD)	F (df1, df2)*	P-value	Partial η^2^
150cm	0cm	411.6 (69.5)	16.426 (2.421, 65.364)	<0.001	0.378
	2cm	398.6 (67.0)			
	3cm	390.0 (69.6)			
	4cm	381.0 (70.2)			
170cm	0cm	689.6 (115.9)	28.011 (2.289, 61.811)	<0.001	0.509
	2cm	680.5 (109.2)			
	3cm	661.4 (102.6)			
	4cm	656.9 (105.2)			

Note. SD = standard deviation; F = F statistics; df = degrees of freedom. *Huynh-Feldt correction to the degrees of freedom due to violation of the sphericity assumption.

**Table III:** Pairwise comparison between leg length discrepancy for second peak force of vertical ground reaction force in the longer leg for children 150cm height between baseline and 2, 3 or 4cm shoe lift.

Shoe lift (cm)	Shoe lift (cm)	Mean difference (95% CI)	SE	P-value
0	2	12.993 (−1.402, 27.389)	5.057	0.096
0	3	21.582 (7.137, 36.026)*	5.074	0.001
0	4	30.561 (13.856, 47.265)*	5.868	<0.001

Note. CI = confidence interval; SE = standard error. *Two pairs were statistically significant: between 0 and 3cm and between 0 and 4cm.

The highest mean second peak force in adults with 170 cm height group occurred during 0cm shoe lift (689.64 N) and declined progressively following the order from 2 (680.54 N) to 3cm (661.44 N) and further down with the 4cm (656.86 N) shoe lift in the longer limb. The analysis showed a significant difference in the mean second peak force between the four levels of shoe lifts (Table II). Pairwise comparisons showed significant reductions in force between 0 and 3cm shoe lift and 0 and 4cm shoe lift, but there was no significant difference in force between 0 and 2cm shoe lift (Table IV).

**Table IV:** Pairwise comparison between leg length discrepancy for second peak force of vertical ground reaction force in the longer leg for adults with 170cm height between baseline and 2, 3 or 4cm shoe lift.

Shoe lift (cm)	Shoe lift (cm)	Mean difference (95% CI)	SE	P-value
0	2	9.102 (−0.850, 19.055)	3.496	0.089
0	3	28.205 (12.467, 43.943)*	5.528	<0.001
0	4	32.778 (19.450, 46.105)*	4.682	<0.001

Note. CI = confidence interval; SE = standard error. *Two pairs were statistically significant: between 0 and 3cm and between 0 and 4cm.

## Discussion

The GRF of the longer leg is different from the short leg^16^. GRF in this study was measured on a simulated longer leg as we wanted to focus on its changes as the simulation amount adjusted. We observed two peaks of vertical GRF during the stance phase. During initial contact, the first peak occurred as the heel got onto the ground, and full weight bearing took place, causing a rapid increase in vertical GRF. In the midstance, vertical GRF dropped slightly as knee flexion unloads the ground. The second peak occurred at the terminal stance as plantar flexors activation pushed the ground, causing a rapid increase in vertical GRF. Finally, the force dropped to zero as the opposite limb took up the body weight. The first and second peaks of vertical GRF were proven to be predictive for quantifying gait asymmetry^5^. The amount of LLD that causing gait asymmetry should be considered for intervention.

Simulated LLD causes changes in the gait pattern similar to patients with real LLDs^17^. In this study, vertical GRF was measured three times for every shoe lifts for both limbs to determine the mean force and strengthen the accuracy of the finding. The GRF was measured and compared in different subjects and limbs to address people's different abilities to adapt to LLDs. This study did not compare the peak vertical GRF between the longer and shorter leg. In this study, we consider the right and left limbs independent due to limb dominancy; in reality, the longer leg may occur in either limb.

This study found that the changes of force at the first peak in children with 150cm and adults with 170cm height volunteers were insignificant. The force created by the body weight without factoring muscle involvement during the initial contact made the difference undetectable. Therefore, we did not perform a pairwise comparison for the first peak force to see the effect of LLD on vertical GRF between the two height groups.

We found that the second peak of GRF on the longer leg gradually reduced as the shoe lift increased. The difference between no LLD and 3cm shoe lift for both height groups became statistically significant. It can be concluded that the minimum discrepancy that caused substantial changes in vertical GRF in the longer leg amongst the normal BMI population of adult patients with a 170cm height was 3cm. This finding is comparable with several other studies. Kaufman *et al*^5^ calculated the asymmetry index using five GRF parameters and found that a more than 2cm discrepancy was significant in causing gait asymmetry. Bhave *et al*^6^, who studied gait parameter improvement, including GRF after lengthening, found no significant difference in GRF between legs after equalisation of limb length within 1cm. Liu *et al*^7^, after performing analysis on the symmetry index with GRF and other parameters, concluded that the acceptable gait symmetry could be observed with a discrepancy of 23.3mm. These findings suggested that the body can compensate for a discrepancy of as small as 2cm.

The amount of LLD to cause a significant difference in the second peak force of GRF in children with 150cm in height was also between 0 and 3cm (2% of body height) or more. This study showed that the amount of LLD that causes a significant change in GRF of the longer leg was similar in children with 150cm and adults with 170cm height groups of volunteers. However, whether similar results can be found in patients of shorter height has yet to be discovered. Thus, the practice of using 3cm as a cut-off point in deciding treatment for children and adults with a height of 150cm and above is proven.

Our study showed that the second peak force of the vertical GRF progressively reduced in the longer leg using the 2, 3, and 4cm shoe lift. Whether a progressive reduction in vertical GRF in the longer leg with an increasing amount of LLD can be inferred as a progressive increment of vertical GRF in the shorter leg is yet to be proven. Many studies show that the shorter leg has a higher GRF^14,18,19^. In terms of biomechanics, greater loading of the short limb should be expected. It is because the step-down difference is more than the opposite during the stance transition from a longer to a shorter limb. Therefore, weight acceptance forces in the shorter limb become higher due to the transfer of body weight from a greater vertical height^20^. The other possible explanation for these findings is related to the stance time. It is reported that, in the presence of LLD, the longer leg will have greater stance time than the shorter leg. Greater stance time contributes to a smaller vertical GRF^21^.

This study has a few limitations. It is not able to answer whether adult with short stature will behave differently. We only measured the point of significant changes from 0 raised in longer leg in the study due to availability of only single force plate in our laboratory. It is recommended for future study to compare two cohorts of height group among adults. The study is best done in a laboratory with two force plate that is capable of measuring both legs at the same cycle.

## Conclusion

Even though adults have better muscle-tendon unit control against the prepubescent group^22^, this study showed that the degree of leg length discrepancy that caused significant changes in second peak ground reaction force in both children with 150 and adults with 170cm height population was similar at 3cm. Therefore, the cut-off point for intervention for both groups are similar with additional consideration of future growth in children. It should be reminded that this study represented a short-term adaptation of the body following acute simulation of LLD.
